# Pharmacological inhibition of myostatin effectively ameliorates osteolytic lesions in syngeneic and xenograft breast cancer mouse models

**DOI:** 10.1038/s41388-025-03622-7

**Published:** 2025-11-17

**Authors:** Julia Reinhardt, Berno Dankbar, Fabienne Geers, Eugenie Werbenko, Christiane Geyer, Annalen Bleckmann, Kerstin Menck, Anne Grözinger, Wolfgang Hartmann, Joke Tio, Carsten Höltke, Anne Helfen, Andreas Lodberg, Rosa Al-Qasemi, Denise Beckmann, Sarah Bödecker, Simon Kleimann, Linda Wessendorf, Deniz Wawersig, Thomas Pap, Corinna Wehmeyer

**Affiliations:** 1 Institute of Musculoskeletal Medicine, University Hospital Muenster, Muenster, Germany; 2 Clinic for Radiology, University Hospital Muenster, Muenster, Germany; 3 Department of Medicine A, Hematology, Oncology, and Pneumology, University Hospital Muenster, Muenster, Germany; 4 West German Cancer Center, University Hospital Muenster, Muenster, Germany; 5 Institute of Pathology, University Hospital Muenster, Muenster, Germany; 6 Department of Gynecology and Obstetrics, University Hospital Muenster, Muenster, Germany; 7 Department of Biomedicine, Aarhus University, Aarhus, Denmark; 8 Department of Clinical Medicine, Aarhus University, Aarhus, Denmark; 9 Department of Endocrinology, Aarhus University Hospital, Aarhus, Denmark; 10 Department of Rheumatology and Immunology, Semmelweis University Budapest, Budapest, Hungary

**Keywords:** Bone cancer, Bone metastases

## Abstract

Breast cancer (BC)-derived bone metastases colonize bone and drive severe bone degradation through complex interactions with bone-resorbing osteoclasts (OCs). Subsequent bone resorption liberates matrix-stored factors, such as TGF-β and calcium, which further stimulate tumor proliferation and exacerbate bone destruction. Myostatin (Mstn), a member of the TGF-β superfamily, is known to enhance OC differentiation and bone resorption in models of musculoskeletal disease; however, its role in BC-associated bone lesions and metastases remains unknown. Here, we demonstrate that bone metastases from BC patients express Mstn, predominantly localized at the osteoclast-rich bone–tumor interface. In vitro, both direct and indirect interactions between BC cells and OC precursors significantly increased OC formation and resorptive activity. Antibody-mediated blockade of Mstn attenuated these effects by inhibiting SMAD2 phosphorylation. In vivo, targeting Mstn in 4T1 and MDA-MB-231 murine models of BC-induced bone destruction resulted in elevated bone density, increased muscle mass, and reduced OC numbers compared to controls. Furthermore, anti-Mstn treatment decreased the burden of bone metastases in MDA-MB-231-bearing mice. Collectively, these findings identify Mstn as a previously unrecognized driver of BC-induced osteolysis and metastases, highlighting its potential as a therapeutic target in metastatic BC.

## Introduction

In breast cancer (BC), the most common cancer in women worldwide, bone is the most common site of metastatic spread leading to significantly reduced patient survival [1]. BC bone metastases affect 65% to 80% of patients leading to the formation of destructive and painful bone lesions which are characterized by increased osteoclastic bone resorption and bone-forming osteoblastic lesions [2]. The bone microenvironment plays a critical role in both osteoclast formation and bone metastases, and many factors are released from the bone matrix as a result of osteoclastic bone resorption, which promotes BC tumor growth and bone metastases progression. In turn, BC tumor cells provide factors that directly or indirectly induce osteoclast formation, mainly by increasing receptor activator of nuclear factor κB ligand (RANKL) expression or enhancing RANKL-mediated signaling [3–6].

This complex crosstalk between BC cells and the bone microenvironment leads to a vicious cycle that further increases both bone destruction and tumor growth [7]. In this context, members of the highly conserved transforming growth factor beta (TGF-β) superfamily, including TGF-βs, activins, inhibins, growth and differentiation factors (GDFs), and bone morphogenetic proteins (BMPs), have been found to play a role in bone metastases and lesions with TGF-β playing the best-established role in osteolytic metastases [8]. Osteoclastic activity in the bone marrow triggered by tumor cells is supposed to result in the release of TGF-β from the bone matrix, which then stimulates cancer cells to release osteolytic cytokines, further promoting bone metastases in BC [7, 9]. Like TGF-β itself, Activin A has been additionally identified as a crucial factor in BC bone metastases, and its inhibition leads to amelioration of the disease [10, 11]. Another member of the TGF-β superfamily is Myostatin (Mstn), also known as GDF-8, which has not been associated with BC bone metastases so far. Mstn is a 25 kDa secreted protein that is expressed primarily in skeletal muscle, which is also its primary target tissue. It has been shown to be a negative regulator of muscle precursors, and inhibition of Mstn improves muscle growth and recovery [12, 13]. It signals through the Activin receptor IIA (ActRIIA) or ActRIIB, with ActRIIB initially identified as myostatin’s prime receptor, to trigger multiple intracellular signaling cascades including the SMAD2/3 and mitogen-activated protein kinase (MAPK) pathways [14]. Deletion of the Mstn gene in mice leads to muscle hypertrophy and hyperplasia with an approximate doubling of muscle mass [13]. Additionally, Mstn deficiency affects fracture healing [15] and bone strength [16, 17] suggesting that Mstn also plays a role in bone formation.

Moreover, increasing evidence indicates that in addition to regulating skeletal muscle growth, Mstn may play a role in many pathological processes, such as obesity/metabolic syndrome [18, 19] and cardiovascular and chronic kidney disease [20]. In line with this, we previously demonstrated that Mstn strongly promotes RANKL-mediated osteoclast formation and that deficiency or pharmacological inhibition of Mstn significantly improves disease severity and especially bone erosion in mice with acute and chronic arthritis [21].

With respect to cancer, Mstn expression is upregulated in the muscle of animals with tumor-induced cachexia and blockage using a soluble form of its receptor (sActRIIB) prevents muscle atrophy and increases survival [22, 23]. Although Mstn is postulated to be a key determinant of muscle loss and cachexia in cancer, there are very few data indicating a role for Mstn in regulating tumor growth and bone metastases in BC. In this regard, the group of Wallner et al. reported a dramatically greater expression of follistatin (FST), Mstn and GDF-11 in low-grade BC (G1) than in benign fibroadenoma but the expression decreased with increasing BC grade [24]. However, the increased expression was associated with a higher overall survival rate in BC patients, which at first glance indicates a beneficial role for Mstn. But taken into account, that FST is a natural antagonist of Mstn, the simultaneously high levels of FST may neutralize Mstn action. Similarly, low FST expression is associated with poor prognosis in patients with triple-negative BC (TNBC) [25] and overexpression of FST in a mouse model of HER2/Neu-induced metastatic BC has a huge inhibitory effect on metastases [26].

Thus, we assessed the role of Mstn in the formation of bone metastases in vivo through pharmacological inhibition of Mstn and analyzed how tumor cell-derived Mstn affects osteoclast formation and activity in vitro. Overall, this study confirms for the first time that Mstn is a novel bone lesion-promoting factor in BC and that blocking Mstn may be a promising treatment option for cancer-mediated bone metastases.

## Results

### Mstn expression in bone metastases of BC patients

**Fig. 1:**
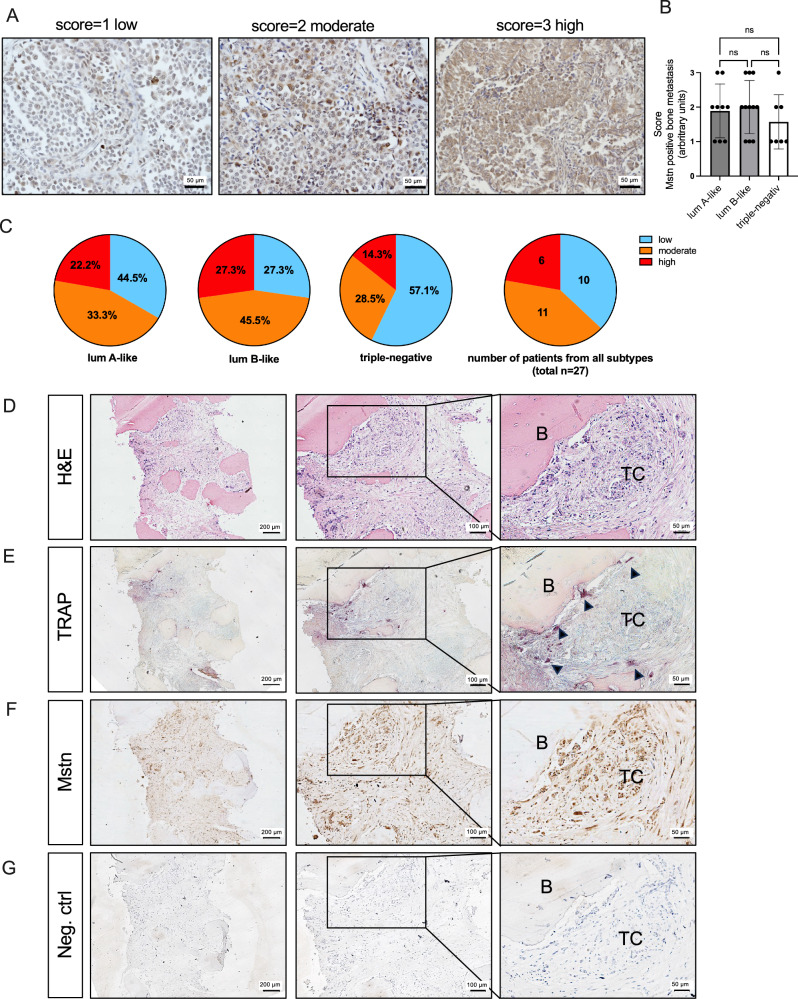
Breast cancer bone metastases is Mstn-positive and located in the osteoclast-bone-tumor interface. **A**, **B** Mstn expression was analyzed in breast cancer bone metastasis from 27 patients. Sections were scored for Mstn expression score 1 = low, score 2 = moderate, score 3 = high. **C** Proportion of low, moderate and high myostatin-positive tissue samples is shown for luminal A-like (lumA-like), luminal B-like (lumB-like), and triple-negative and all subtypes. Luminal A-like, *n* = 9; luminal B-like, *n* = 11 and triple-negative, *n* = 7; All, *n* = 27; All data are mean ± SD, one-way ANOVA, Kruskal-Wallis test with multiple comparisons, *p ≤ 0.05, **p ≤ 0.01. **D**–**G** Human iliac crest biopsy sections of bone metastases from TNBC patients (*n* = 7). **D** H&E-overview staining (**E**) TRAP+ osteoclasts and (**C**) Mstn expression (brown) (**G**) Negative control:IgG. B = bone, TC = tumor cells, arrowheads = osteoclasts.

To gain initial insight into the role of Mstn in BC, we determined the overall positivity rate of Mstn in a cohort of 27 BC patients with histologically confirmed bone metastases. Immunohistochemical evaluation revealed that all investigated tissue biopsies with bone metastases were Mstn-positive. Among these, 17 cases exhibited moderate to high Mstn expression, while 10 cases showed low expression levels. Notably, we detected subtype-specific differences: Mstn expression was more frequently moderate to high in the luminal B subtype (72.8%), compared to luminal A (55.5%) and TNBC (42.8%) (Fig. 1A–C).

To investigate whether the pathological effects of Mstn on bone are predominantly mediated by its local secretion within the bone microenvironment by bone-metastatic tumor cells, rather than through systemic elevation, we performed an additional analysis comparing circulating Mstn levels among three patient groups: those with primary BC tumors only, those with both primary BC tumors and bone metastases, and control patients harboring non-invasive proliferative lesions, including lobular carcinoma in situ (LCIS) and ductal carcinoma in situ (DCIS). Our findings demonstrated that circulating Mstn levels are comparable across these groups (Supplementary Fig. 1A), suggesting that the presence of primary tumor cells, either alone or accompanied by bone metastases, does not significantly impact systemic Mstn concentrations relative to non-metastatic controls. These data support the notion that the pathological effects of Mstn are likely driven by its local production within the bone microenvironment, rather than by a systemic increase.

For a more detailed examination of the tumor-cell osteoclast niche in BC patients with bone metastases, we performed hematoxylin–eosin (H&E) staining, tartrate-resistant acid phosphatase (TRAP) staining, and Mstn immunostaining on biopsies obtained from the posterior iliac crest. In representative sections, we observed bone metastatic lesions characterized by infiltration of tumor cells into the medullary cavity, largely replacing normal bone marrow components (Fig. 1D). Furthermore, TRAP staining revealed numerous osteoclasts, particularly localized at the bone–tumor interface, where Mstn-positive metastases were detected (Fig. 1E–G).

Taken together, these findings indicate that tumor cells within human bone metastases consistently express Mstn, suggesting that BC-derived Mstn may play a pivotal role in driving the formation and progression of bone lesions.

### 4T1 BC cell-derived Mstn promotes OC differentiation and resorption in vitro

**Fig. 2:**
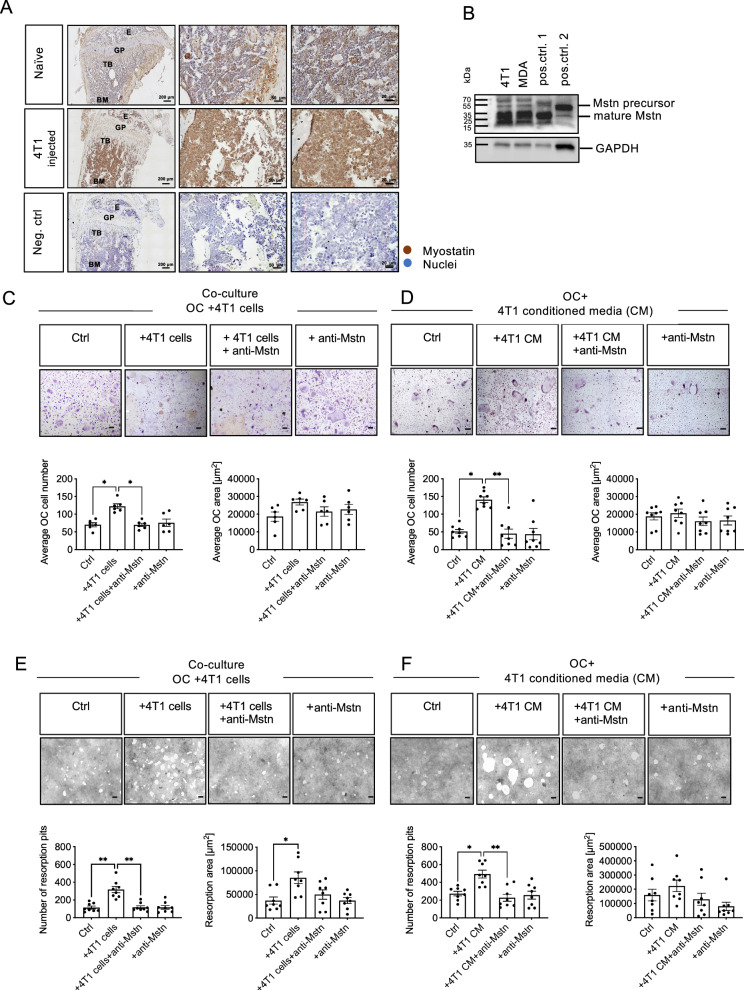
Enhanced OC differentiation and bone resorption by 4T1 BC-cell OC interaction is mediated by Mstn in vitro. **A** Mstn expression in naïve (healthy control) and in tibiae 14 days after 4T1-luc2 tumor cell injection. E: epiphysis, GP: growth plate, TB: trabecular bone, BM: bone marrow. **B** Representative Western Blot of Mstn in 4T1, MDA-MB-231 breast cancer cell lines. Positive controls: pos. ctrl1: osteocyte cell line MLO-Y4, pos. ctrl2: mouse skeletal muscle tissue lysate. **C** OC differentiation in co-cultures of WT BMDMs and 4T1 tumor cells (*n* = 6) and anti-myostatin antibody (5 µg/ml). **D** OC differentiation of WT BMDMs cultured with 10% conditioned medium of 4T1 tumor cells and anti-myostatin antibody (*n* = 8). **C**, **D** TRAP staining was used to visualize mature OCs. **E** WT BMDMs were co-cultured with 4T1 tumor cells on calcium phosphate coated plates and stimulated with anti-Mstn antibody (5 µg/ml) (*n* = 8). **F** WT BMDMs were cultured on calcium phosphate coated plates and treated with 10% conditioned medium of 4T1 tumor cells and 5 µg/ml anti-Mstn (*n* = 8). **C**–**F** All cells were cultivated in the presence of 30 ng/ml M-CSF and 50 ng/ml RANKL. Control: differentiated WT BMDMs only. All data are mean ± SEM, one-way ANOVA, Kruskal-Wallis test with multiple comparisons, *p ≤ 0.05, **p ≤ 0.01; scale bar 200 μm.

Consistent with the expression data of human BC patients, we also detected Mstn expression in bone metastases induced by intracardiac injection of murine 4T1 BC cells into mice. In contrast to tibial sections of healthy mice, sections of 4T1-injected animals exhibited more intense staining, indicating strong Mstn expression by infiltrated 4T1-BC cells (Fig. 2A). In line with these data, in vitro cultivated 4T1 BC cells also express Mstn (Fig. 2B).

To investigate the influence of BC cell-derived Mstn on osteoclast differentiation, wild type (WT) BMDMs were co-cultured with the murine BC cell line 4T1 or cultured with 4T1-derived conditioned medium (CM) alone or in combination with a neutralizing Mstn antibody. Additionally, co-cultures were stimulated with M-CSF and RANKL followed by TRAP-staining to detect mature, multinucleated osteoclasts (Fig. 2C, D).

Co-culture of OCs and 4T1 cells led to a significant increase in the number of OC (4T1 cells: 1.7-fold) compared to that in the OC control group. Most interestingly, treatment of the co-culture with an anti-Mstn antibody completely abrogated the 4T1-mediated effect on OC differentiation (4T1 cells + anti-Mstn). OC size was not altered in any of the settings (Fig. 2C). To investigate whether this effect on osteoclastogenesis by cancer cells is dependent on direct cell-cell contact, OCs were cultured with CM of 4T1 cells. Similarly, significantly more OCs could be detected as a result of stimulation with 4T1 CM (4T1 CM: 2.9-fold) than in the control group, which could be again effectively prevented by addition of the anti-Mstn antibody (4T1 CM + anti-Mstn). The OC size was not altered (Fig. 2D). These results strongly indicate that murine BC cells are able to promote osteoclast differentiation even without direct cell-cell contact by secreting soluble factors such as Mstn.

Since BC-derived Mstn enhanced RANKL-mediated OC formation, we next investigated whether OC resorption was also influenced. Both direct cultivation of WT BMDMs with 4T1 cells (Fig. 2E) or indirect stimulation with 4T1 CM (Fig. 2F) resulted in a significantly greater number of resorption pits, probably due to greater OC numbers (4T1 cells: 2,8-fold, 4T1 CM: 1,8-fold). In addition, a significantly greater resorbed area was observed when BMDMs were co-cultured with 4T1 cells (2.3-fold), while a trend was observed only after stimulation with 4T1 CM (1.4-fold). For both experimental approaches, the addition of the anti-Mstn antibody resulted in a significantly reduced number of resorption pits compared to those in the controls, respectively (4T1 cells: -70%; 4T1 CM:-54%) and a reduction in the resorbed area (4T1 cells: -42%; 4T1 CM: -42%).

### MDA-MB-231 BC cell-derived Mstn promotes OC differentiation and bone resorption in vitro

To investigate whether this effect can be translated to human BC similar experiments were performed with the metastatic human TNBC cell line MDA-MB-231 (MDA). For this purpose, a xenograft model was generated by injecting MDA cells into the caudal artery of immunodeficient NSG mice.

**Fig. 3:**
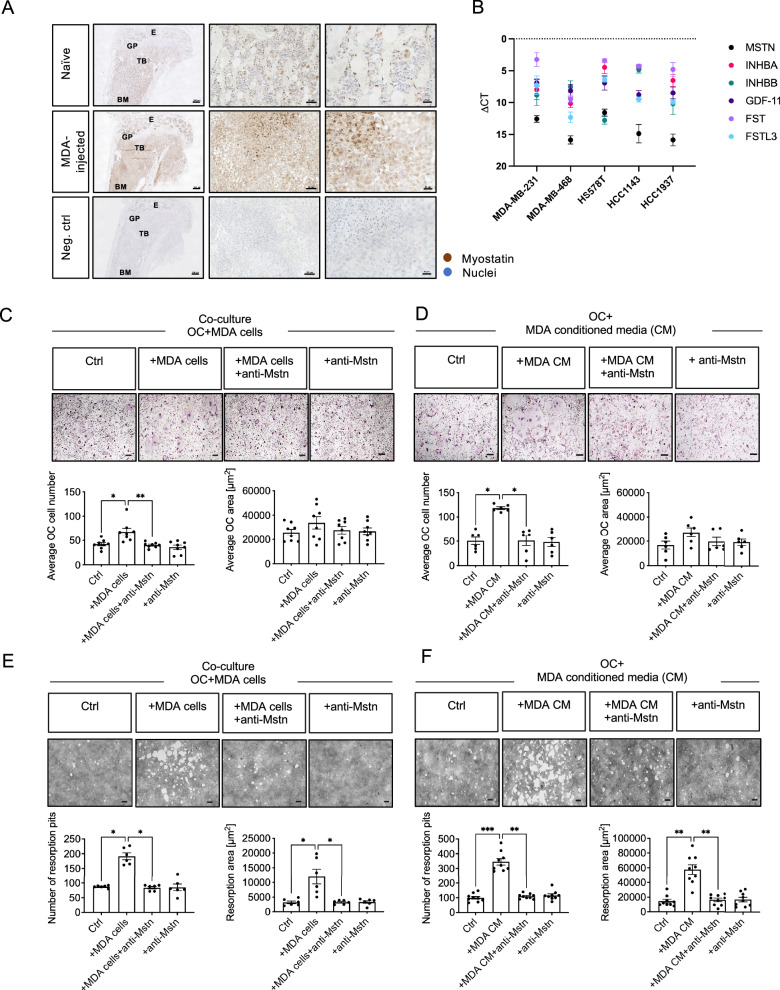
Enhanced OC differentiation and bone resorption by MDA BC-cell OC interaction is mediated by Mstn in vitro. **A** Mstn expression in naïve (healthy control) and in tibiae 21 days after MDA-MB-231-luc tumor cell injection. E: epiphysis, GP: growth plate, TB: trabecular bone, BM: bone marrow. **B** RNA expression of different GDFs and their respective antagonists in human triple negative breast cancer cell lines. qPCR analysis of MSTN (Myostatin), INHBA (Activin A) INHBB (Activin B), GDF-11 (Growth and Differentiation Factor 11), FST (Follistatin) and FSTL3 (Follistatin-like 3). **C** OC differentiation in co-cultures of WT BMDMs and MDA-MB-231 cells (*n* = 8) treated with anti-myostatin antibody (5 µg/ml). **D** OC differentiation of WT BMDMs cultured with 10% conditioned medium of MDA-MB-231 BC cells and anti-Mstn antibody (*n* = 6). **E** WT BMDMs were co-cultured with MDA-MB-231 cells on calcium phosphate coated plates and stimulated with anti-Mstn antibody (*n* = 8). **F** WT BMDMs were cultured on calcium phosphate coated plates and treated with 10% conditioned media of MDA-MB-231 tumor cells and anti-Mstn antibody (*n* = 8). **B**–**E** All cells were cultivated in the presence of 30 ng/ml M-CSF, 50 ng/ml RANKL. Control: differentiated WT BMDMs only. All data are mean ± SEM, one-way ANOVA, Kruskal-Wallis test with multiple comparisons, *p ≤ 0.05, **p ≤ 0.01; scale bar 200 μm.

In accordance with the expression data of human BC patients and of the syngeneic 4T1 tumor model, expression of Mstn was also detected in bone metastases of the MDA xenograft tumor model. In contrast to tibiae sections from healthy mice, sections from MDA-injected animals showed elevated staining, indicating that Mstn was also expressed by infiltrated MDA cells (Fig. 3A). In line with these data, in vitro cultivated MDA BC cells also express Mstn (Figs. 2B, 3B). To determine whether Mstn and other members of the TGF-β superfamily, along with their respective antagonists, are exclusively expressed in 4T1 and MDA-MB-231 cells, we extended our analysis to a broader panel of human TNBC cell lines. Specifically, we assessed the expression levels of Mstn (MSTN), Activin A (INHBA), Activin B (INHBB), GDF-11, and the known antagonists FST and follistatin-like 3 (FSTL3). Our analysis revealed that all tested TNBC cell lines expressed these TGF-β superfamily members and their antagonists (Fig. 3B), suggesting that their expression is not restricted to MDA-MB-231 cells but may represent a broader characteristic of TNBC biology. Moreover, the skeletal muscle, that is the primary physiological source of Mstn could also contribute to enhanced Mstn levels during BC metastasis influencing OC formation. Therefore, we analyzed Mstn expression in skeletal muscles located in close anatomical proximity to bone lesions in both the 4T1 murine BC model and the MDA-MB-231 human BC model, relative to naïve/healthy controls (Supplementary Fig. 2A–C). Our analysis revealed no significant alterations in Mstn levels within these muscles, indicating that local production of Mstn by adjacent skeletal muscle does not influence osteoclastogenesis in these BC models.

Similar to 4T1 cells, co-culture of BMDMs with MDA cells led to a significant increase in the number of OCs (MDA cells: 1.6-fold) (Fig. 3C). Simultaneous stimulation of OCs with MDA cells and an anti-Mstn antibody abrogated of the BC cell-mediated stimulatory effect. Similarly, stimulation of WT BMDMs with MDA CM significantly increased the number of OCs (MDA CM: 2.3-fold), and the blockade of Mstn completely inhibited the MDA-mediated stimulation of OC differentiation (Fig. 3D), indicating that human MDA BC cells are also able to influence OC differentiation via the secretion of Mstn.

Furthermore, stimulation of OCs with MDA cells (Fig. 3E) or MDA CM (Fig. 3F) led to a significant increase in both the number of resorption pits (MDA cells: 2.2-fold; MDA CM: 3.5-fold) and the total resorbed area (MDA cells: 3.7-fold; MDA CM: 4-fold). Again, the MDA-mediated increase in both the number of resorption pits and the resorption area was completely prevented by anti-Mstn antibody. Taken together, these results suggest that Mstn from human BC cells, as well as from murine BC cells, promotes OC differentiation and bone resorption.

### Anti-Mstn treatment does not affect tumor spreading in a syngeneic BC model

Since BC cell-derived Mstn appears to play an important role in OC differentiation and resorption, we investigated the effect of Mstn on BC tumor progression in vivo. For this purpose, a syngeneic mouse model with murine 4T1 BC cells was established. Because the development of bone metastases rather than that of the primary tumor was to be investigated, 4T1 cells labelled with luciferase were injected into the left ventricle of 4-week-old female BALB/c mice, resulting in rapid spread of cancer cells throughout the body and excluding the formation of a primary tumor. After a recovery period of 4 days, the animals were treated with vehicle (PBS) or with the neutralizing anti-Mstn antibody RK35 (Pfizer) every second day.

**Fig. 4:**
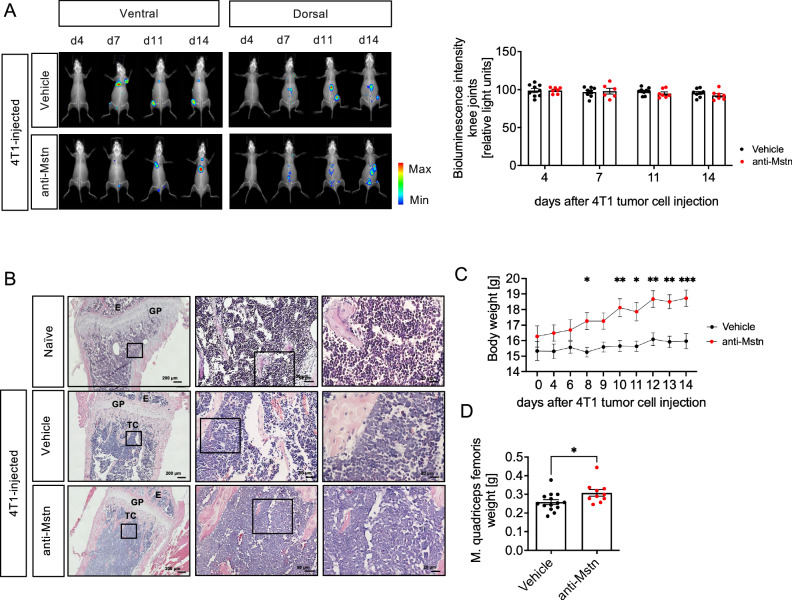
Effect of anti-Mstn treatment on tumor progression and body weight in a syngeneic 4T1 mouse model of BC metastases. **A** Representative bioluminescence images of 4T1 luc2+ cells injected mice at the indicated time points from ventral (left) and dorsal (right) views. Quantitative evaluation of bioluminescence intensity in knee joints of 4T1 luc2 + -injected and vehicle (*n* = 9) or anti-Mstn treated mice (*n* = 7). **B** Representative H&E-staining of tibia from naïve Balb/c mice (healthy control) and mice injected with 4T1 luc2+ breast cancer cells (E = epiphysis, GP = growth plate, TC = tumor cell). **C** Effect of anti-Mstn treatment on body weight of 4T1 luc2 + -injected mice (vehicle: *n* = 7, anti-Mstn: *n* = 5). **D** Effect of anti-Mstn treatment on quadriceps femoris muscle weight of 4T1-injected mice (vehicle: *n* = 14, anti-Mstn: *n* = 10, left and right). Shown are individual data and mean values ± SEM, one-way ANOVA, Kruskal-Wallis test with multiple comparisons or Mann-Whitney test, two-tailed, *p ≤ 0.05, **p ≤ 0.01, ***p ≤ 0.001.

To investigate the impact of Mstn inhibition on the distribution of luciferase labelled 4T1 tumor cells, bioluminescence (BLI) intensities were evaluated in knee joint areas. As shown in Fig. 4A, beginning on day 7 after 4T1 luc+ injection, the first BLI signals were detected in hind legs of these animals, especially around the knee joint. The distribution of cancer cells was not only limited to hind limbs, but could also be detected in the spine or other organs such as kidney. Interestingly, evaluation of BLI signals in knee joints revealed no differences in BLI intensities between antibody-treated and untreated animals. In accordance with the BLI data, very densely packed areas of tumor cells, preferentially located below the growth plate, could be observed in tibiae of tumor-bearing mice (Fig. 4B). These metastatic areas could be clearly distinguished from normal bone marrow. Again, no differences were detected between RK35-treated and untreated animals, suggesting that Mstn plays no or only a minor role in bone metastases formation in this model.

As Mstn is known to be a negative regulator of muscle growth and its deletion leads to muscle hypertrophy, body weight was measured throughout this study and the weight of the musculus quadriceps femoris was analysed at the end of the study. Antibody-treated tumor mice showed a significantly increased body weight from day 10 after cancer cell injection compared to vehicle-treated tumor mice (Fig. 4C). Moreover, treatment of tumor-bearing mice with anti-Mstn antibody led to an increase in quadriceps femoris weight, indicating an effective inhibition of Mstn by anti-Mstn antibody RK35 (Fig. 4D).

### Anti-Mstn treatment effectively reduces OC formation and bone lesions in a syngeneic BC model

4T1-injected mice were examined not only histologically but also morphometrically using µCT reconstruction to assess bone alterations. For analysis of trabecular bone, a defined area below the growth plate was analyzed in healthy as well as in 4T1-injected tumor-bearing mice.

**Fig. 5:**
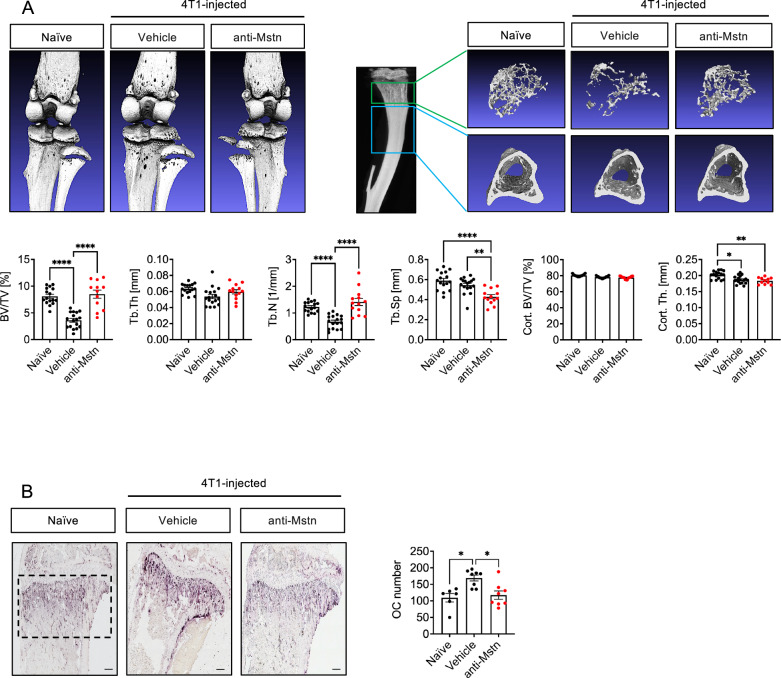
Anti-Mstn treatment effectively reduces bone lesions and OC formation in a syngeneic 4T1 mouse model of BC metastases. **A** Effects of anti-Mstn antibody treatment on trabecular and cortical bone parameters. Shown are representative images of a defined area of μCT-reconstructed tibiae. The tibiae of naïve (healthy control) (*n* = 8), anti-Mstn-treated (*n* = 7) and vehicle mice (*n* = 9) were analyzed for trabecular bone volume fraction (BV/TV, %), trabecular thickness (Tb. Th, mm), trabecular number (Tb.N, 1/mm) and trabecular separation (Tb.Sp., mm) as well as cortical bone fraction (Cort. BV/TV, %) and cortical thickness (Cort. Th, mm) using the BRUKER μCT CTAn software. Left and right tibiae were analyzed. **B** TRAP-positive OCs in tibiae sections of naïve (healthy control) (*n* = 3), vehicle (*n* = 4) and anti-Mstn-treated mice (*n* = 4). Shown are individual values and mean ± SEM, one-way ANOVA, Kruskal-Wallis test with multiple comparisons, *p ≤ 0.05, **p ≤ 0.01, ***p ≤ 0.001, ****p ≤ 0.0001. Scale bar 200 μm.

Differences in trabecular density were already visible in the reconstructed images of tibiae, with a distinctly lower number of trabeculae in 4T1-injected mice than in healthy control mice. Notably, treatment with anti-Mstn antibody significantly improved the loss of trabecular structures (Fig. 5A). To quantify these changes, different trabecular parameters such as bone volume/ tissue volume (BV/TV, %), trabecular number (Tb.N, 1/mm), trabecular thickness (Tb.Th, mm), and trabecular separation (Tb.Sp, mm) were evaluated. Quantification revealed a significant decrease in bone volume fraction as well as trabecular numbers in 4T1-injected mice compared to those in healthy control mice (-56% and -48%, respectively). Most importantly, treatment with anti-Mstn antibody significantly improved these bone parameters to levels approximating those of tumor-free healthy mice. In contrast, anti-Mstn antibody treatment did not improve cortical bone density parameters.

Antibody-mediated prevention of trabecular bone loss seems to be due to a decreased number of OCs, as shown by a distinct reduction in the number of TRAP-positive cells in tibiae of antibody-treated mice (Fig. 5B). Quantification revealed significantly more OCs in tibiae of 4T1-injected animals than in those of healthy controls (109 vs. 169 OCs) and a significantly reduced number of OCs, comparable to that in healthy controls, in antibody-treated 4T1-injected animals. These findings highly consistent with our in vitro data demonstrating that OC differentiation is inhibited by Mstn blockade. Taken together, these data strongly suggest that Mstn is an important factor in the interaction between cancer cells and OCs and thus in cancer-induced osteolysis.

### Anti-Mstn treatment affects tumor spreading in a xenograft BC model

To additionally investigate the effect of Mstn in a xenograft BC mouse model, human MDA-MB-231-Luc+ cells were injected into the caudal artery of 4-week-old female NSG mice, leading to the formation of bone metastases, especially in hind limbs. Starting on day 7, infected mice were treated with vehicle (PBS) or an anti-Mstn antibody twice weekly.

**Fig. 6:**
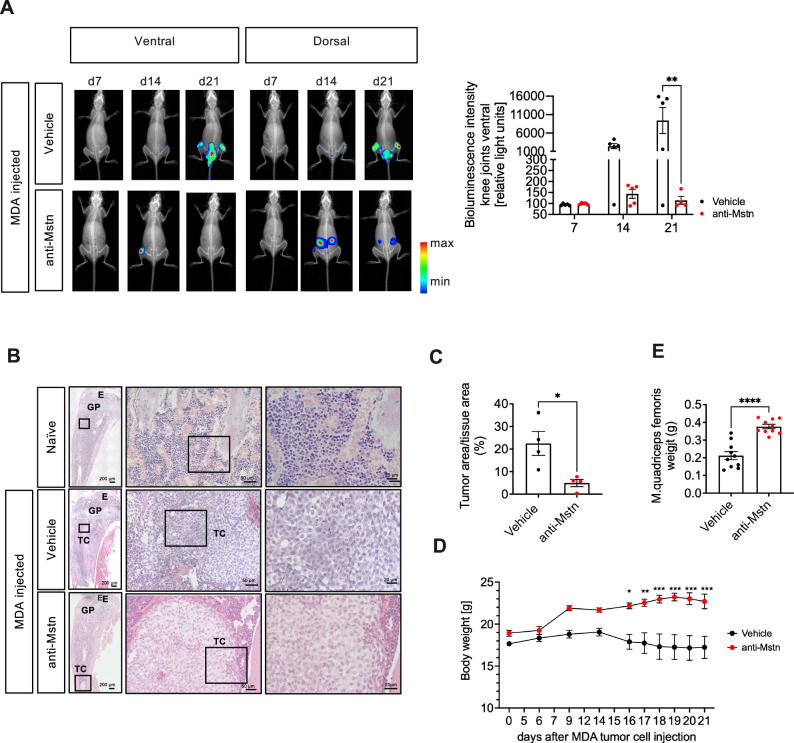
Effect of anti-Mstn treatment on tumor progression and body weight in a xenograft MDA mouse model of BC metastases. **A** Representative bioluminescence images of MDA-MB-231-luc cells injected mice at the indicated time points from ventral (left) and dorsal (right) views. Quantitative evaluation of bioluminescence intensity in knee joints of MDA-MB-231-luc-injected and vehicle (*n* = 5) or anti-Mstn treated mice (*n* = 5). **B** Representative H&E-staining of tibiae from naïve Balb/c mice (healthy control) and mice injected with MDA-MB-231-luc breast cancer cells (E= epiphysis, GP= growth plate, TC= tumor cell). **C** Quantification of tumor cells in tibiae sections of MDA-MB-231-luc-injected mice in anti-Mstn or vehicle treated controls. n = 4 each group. **D** Effect of anti-Mstn treatment on body weight of MDA-MB-231-luc -injected mice (vehicle: *n* = 5, anti-Mstn *n* = 5). **E** Effect of anti-Mstn treatment on quadriceps femoris muscle weight of MDA-MB-231-luc-injected mice (vehicle: *n* = 10, anti-Mstn: *n* = 10, left and right). Shown are individual data and mean values ± SEM, one-way ANOVA, Kruskal-Wallis test with multiple comparisons or Mann-Whitney test, two-tailed, *p ≤ 0.05, **p ≤ 0.01, ***p ≤ 0.001.

To track the growth and metastases of MDA-MB-231-Luc+ cells in these mice, BLI imaging was performed. Representative images clearly showed that human BC cells became increasingly distributed throughout the hind limbs, spine and ovaries of the injected mice during the course of this study. Interestingly, BLI intensities were significantly lower in the knee joints of anti-Mstn treated mice than in those of vehicle control mice (Fig. 6A), confirming that Mstn seems to play a role in bone metastases in this model.

To verify whether cancer cells had metastasized into the bone, tibiae sections were stained with H&E (Fig. 6B). Interestingly, tumor cells of anti-Mstn treated mice accumulated only in small areas of the bone marrow compared to vehicle controls, supporting the notion that this antibody also suppress metastatic tumor growth in bones (Fig. 6C).

While the body weight of vehicle-treated mice decreased slightly during the course of the study and was even slightly below the initial weight at the end, antibody-treated mice continued to gain body weight, so that they weighed significantly more than vehicle-treated mice at the end of the experiment (Fig. 6D). Furthermore, the significantly greater weight of the quadriceps femoris in antibody-treated tumor mice again demonstrated an effective inhibition of Mstn (Fig. 6E).

### Anti-Mstn treatment effectively reduces OC formation and bone lesions in a xenograft BC model

**Fig. 7:**
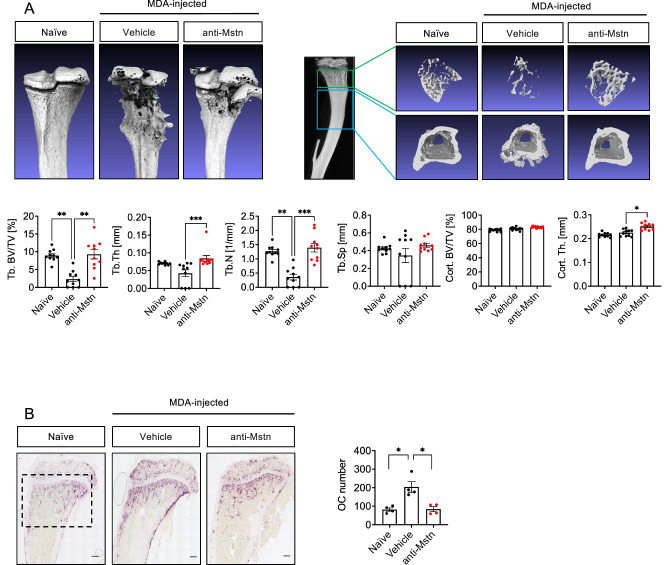
Anti-Mstn treatment effectively reduces bone lesions and OC formation in a xenograft MDA mouse model of BC metastases. **A** Effects of anti-Mstn antibody treatment on trabecular and cortical bone parameters. Shown are representative images of a defined area of μCT-reconstructed tibiae. The tibiae of naïve (healthy control) (*n* = 5), anti-Mstn-treated (*n* = 5) and vehicle mice (*n* = 5) were analyzed for trabecular bone volume fraction (BV/TV, %), trabecular thickness (Tb. Th, mm), trabecular number (Tb.N, 1/mm) and trabecular separation (Tb.Sp., mm) as well as cortical bone fraction (Cort. BV/TV, %) and cortical thickness (Cort. Th, mm) using the BRUKER μCT CTAn software. Left and right tibiae were analyzed. **B** TRAP-positive OCs in tibiae sections of control (*n* = 4), vehicle (*n* = 4) and anti-Mstn-treated mice (*n* = 4). Shown are individual values and mean ± SEM, one-way ANOVA, Kruskal-Wallis test with multiple comparisons, *p ≤ 0.05, **p ≤ 0.01, ***p ≤ 0.001, ****p ≤ 0.0001. Scale bar 200 μm.

µCT analysis of tibial bones of MDA-injected mice revealed strong bone destruction reflected by heavily destroyed trabecular structures. This tumor-mediated bone loss was substantially improved by treatment with the anti-Mstn antibody, as shown by an increase in the amount of trabecular bone in treated tumor-bearing mice (Fig. 7A).

Quantification of different trabecular bone parameters, BV/TV, Tb.Th, and Tb.Sp revealed a significant decrease in bone volume as well as Tb.N in MDA-injected mice compared to healthy control mice (−74% and −70%), respectively. Remarkably, treatment with anti-Mstn antibody significantly improved these bone parameters to levels approximating those of tumor-free healthy mice. Anti-Mstn antibody treatment did not improve cortical BV/TV parameters but slightly increased cortical thickness.

Again, the decrease in trabecular bone appeared to be due to fewer OCs, as shown by the reduced number of TRAP-positive cells in tibiae of antibody-treated mice. Similar to those in 4T1 tumor-bearing mice, quantification revealed significantly more OCs in tibiae of MDA-injected animals compared to healthy controls (82 vs. 204 OCs), and a significantly reduced number of OCs, down to level of naïve mice, in antibody-treated MDA-injected mice (Fig. 7B). These data further support our hypothesis that Mstn plays an important role in cancer-induced bone metastases.

### Anti-Mstn treatment reduces tumor-cell mediated SMAD-2 phosphorylation

**Fig. 8:**
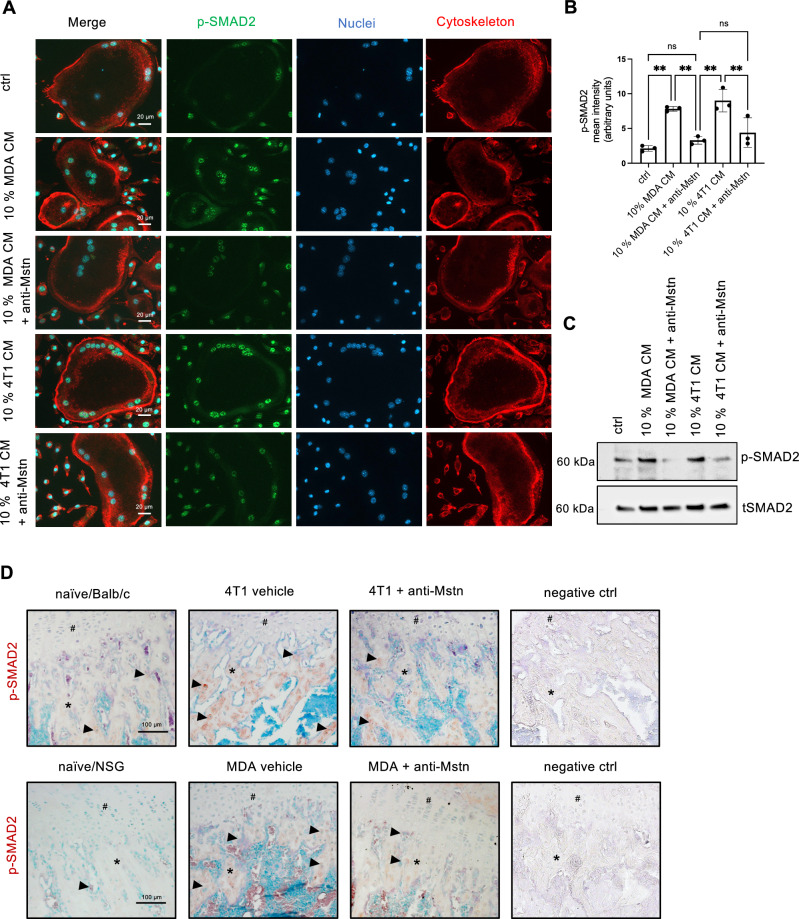
Anti-Mstn treatment reduces tumor-cell mediated SMAD2 phosphorylation in osteoclasts. **A** p-Smad2 IF staining in in vitro differentiated WT OC cultured with 10% conditioned medium of 4T1 or MDA-MB-231 tumor cells and anti- Mstn antibody (5 µg/ml). green: p-SMAD2, red: phalloidin/cytoskeleton, blue: DAPI/nuclei. **B** Quantification of p-SMAD2 signal intensity in vitro differentiated WT OC using Fiji software. Data represent mean values from five different regions per sample (*n* = 3 WT mice per group). **C** Representative Western Blot of p-SMAD2 in in vitro differentiated WT OC cultured with 10% conditioned medium of 4T1 or MDA-MB-231 tumor cells and anti-Mstn antibody. **D** Representative immunostaining of p-SMAD2 in femora sections from naïve mice, 4T1 and MDA tumor-bearing mice treated with or without anti-Mstn. *n* = 3; red: p-SMAD2; green: nuclei; arrow head: p-SMAD2 positive osteocytes and osteoclasts; asterix: trabecular bone; hash: growth plate; negative control: IgG. All data are mean ± SD, one-way ANOVA, Kruskal-Wallis test with multiple comparisons, *p ≤ 0.05, **p ≤ 0.01.

To further investigate the effect of BC tumor cells on SMAD signaling, we performed immunostaining for phosphorylated SMAD2 (p-SMAD2) in in vitro differentiated WT OCs treated with 10% CM derived from 4T1 or MDA cells (Fig. 8A). The immunostaining demonstrated that exposure to tumor cell-derived CM significantly increased SMAD2 phosphorylation in OCs (4T1 CM: 4.3-fold increase; MDA CM: 3.7-fold increase). Notably, co-incubation with anti-Mstn antibody markedly attenuated this effect (Fig. 8B).

These findings were further corroborated by Western blot analysis of OC lysates, which similarly showed enhanced p-SMAD2 levels upon CM treatment and their reduction following anti-Mstn treatment (Fig. 8C). Additionally, to validate these observations ex vivo, p-SMAD2 expression was analyzed by immunohistochemical staining on femur sections. Consistent with the in vitro data, anti-Mstn treatment effectively reduced tumor cell-induced SMAD2 phosphorylation in bone tissue (Fig. 8D).

## Discussion

Bone is the most common site of distant metastases in patients with metastatic BC leading to high morbidity and mortality due to excessive tumor related and OC-mediated bone resorption [1, 27]. In particular, patients with TNBC have a poor prognosis and are associated with early and very aggressive metastases and a high recurrence rate within the first 3 years [28].

Bone-modifying agents, including bisphosphonates and denosumab, are commonly prescribed for patients with BC bone metastases to delay skeletal-related events and to reduce pain. However, these treatments do not completely prevent pathological fractures. New bone-targeted and effective therapies are needed to stop metastatic bone degradation and increase bone strength to improve quality of life.

During metastases, disseminated BC cells settle in “pre-metastatic niches” in the bone matrix and interact with surrounding cells by releasing pro-osteoclastogenic factors resulting in local bone damage. However, the exact composition of these metastatic niches and factors that lead to formation of metastatic growth and associated osteolytic lesions are still unclear and need further investigation.

Members of the TGF-β superfamily such as Activin A and their signaling pathways have been closely linked to tumor progression [29–35]. It has been reported that elevated Activin A expression in high-grade breast tumor tissue positively correlates with the number of skeletal metastases and is associated with poorer prognosis and metastases-free survival in BC patients [31, 36].

Unlike Activin A, the TGF-β superfamily member Mstn has so far not been linked to tumor progression. In this study, we found for the first time high Mstn expression in bone metastases from patients with luminal A, luminal B and TNBC. HER2-enriched subtypes were not investigated. Luminal A and luminal B xenograft models rarely spontaneously metastasize to bone in mice. In contrast, more aggressive bone-tropic TNBC models such as 4T1 and MDA-MB-231 readily develop spontaneous bone metastases, making them widely established systems for studying the natural progression of bone metastasis. For this reason, we employed these models to investigate the role of Mstn in bone metastasis. We demonstrated that *Mstn* is highly expressed in both the 4T1 syngeneic and the MDA xenograft BC mouse models.

For decades, increased bone resorption in metastatic BC has been known to be mediated mainly by OCs. In support of this notion, a study by Taube et al. that analyzed bone biopsies from women with BC and osteolytic bone metastases using histomorphometry. They reported that the ratio of OCs to eroded surface area in tumor-affected bone regions was significantly greater than that in sites distant from tumor tissue [37]. Together, these findings suggest that the increased bone resorption in BC is mainly mediated by OCs that are locally activated by tumor-secreted factors. Considering this assumption and the fact that Mstn directly promotes OC-mediated bone destruction in rheumatoid arthritis mouse models [21], we wondered whether Mstn is one of these pro-osteoclastogenic factors responsible for BC bone lesions.

In this study, we confirmed that BC tumor cells increase osteoclastogenesis and OC-mediated bone resorption in vitro, independent of direct cell-cell contact, suggesting that the release of secreted factors has an impact on OC formation. Previous studies reported that BC patients have higher levels of phospho-SMAD2 and phospho-SMAD3 than healthy individuals, indicating that SMAD signaling is highly active in these tumors [38]. Like other TGF-β superfamily members, Mstn has been linked to the activation of the SMAD signaling pathway [39–41]. Thus, BC cell-derived Mstn likely binds to its heterodimeric receptor complex ActRIIA or IIB on the surface of progenitor cells and mature OCs, leading to subsequent activation of SMAD2 and ultimately to the expression of several OC-specific target genes. Accordingly, Mstn can stimulate RANKL-dependent expression of integrin αvβ3, DC-STAMP, NFATc1 or calcitonin receptors, which are responsible for OC development [21]. Consistent with these findings, our in vitro experiments demonstrated that inhibition of Mstn markedly attenuated tumor cell–induced osteoclastogenesis and bone resorption, effects that were mediated through SMAD2 signaling. Thus, Mstn seems to be a prominent factor in promoting osteoclastogenesis and OC activity in the presence of BC cells.

Further studies reported that pharmacological inhibition of Mstn directly or by decoy receptors improves bone integrity and/or muscle loss in mouse models of osteoarthritis, rheumatoid arthritis, osteogenesis imperfecta and diabetes [21, 42–44]. In consideration of this fact, we tested the anti-Mstn antibody in a 4T1-syngeneic and a MDA-xenograft BC metastases mouse model and clearly demonstrated that anti-Mstn treatment significantly rescued trabecular bone loss and decreased OC formation in both mouse models. Together with the in vitro results showing that anti-Mstn completely blocks tumor cell mediated OC formation and resorption, these data strongly demonstrated that anti-Mstn treatment is protective against bone damage in metastatic BC.

Notably, antibody treatment did not alter tumor growth in the 4T1-model but significantly decreased metastases in the MDA-model. In this regard, intra-cardiac injection of 4T1-luc cells, the current standard method for investigating distant metastases, is associated with widespread colonization of cancer cells in organs other than bone. Additionally, the location and number of metastases can be variable and depend on the cell lines used [45]. In contrast, it has been reported that MDA-cells injected into the tail caudal artery (CA) frequently metastasize to bone, especially to the hind limbs, with fewer fatal metastases in other organs [46]. Considering that BLI signals in the 4T1 model were much lower compared to the MDA model in knee joints, it is possible that a reduction in metastases formation caused by anti-Mstn treatment was not detectable.

Further studies described follistatin, an intrinsic inhibitor of Mstn and Activin A, as a metastases suppressor in a HER2-positive BC mouse model, supporting our hypothesis that antibody-mediated inhibition of Mstn may affect tumor growth in the xenograft BC model [26].

Numerous clinical and preclinical studies have explored the therapeutic potential of targeting the Mstn and/or Activin signaling pathways, primarily in the context of muscular dystrophies, reflecting growing interest in these factors as regulators of musculoskeletal health. However, relatively few investigations have examined these pathways in the context of cancer. Notably, one study assessed the ligand trap ActRIIA-Fc (ACE-011, Sotatercept) in patients with multiple myeloma and osteolytic lesions, demonstrating an improvement in bone mineral density [47]. ActRIIA-Fc functions by sequestering multiple ligands of the TGF-β superfamily, including Activins, Mstn, GDF-11, and select BMPs, and was recently approved by the FDA for the treatment of pulmonary arterial hypertension in adults (NCT04576988).

In contrast, anti-myostatin antibody, which selectively neutralize Mstn, are currently undergoing clinical evaluation in patients with muscle-wasting disorders (e.g., NCT06877689), but have not yet received regulatory approval for clinical use.

Collectively, our results clearly show that selective neutralizing Mstn reduces the development of metastases, osteoclasts and bone damage. Therefore, blocking Mstn might be a promising treatment of cancer-associated osteolytic lesions.

## Methods

### Human bone tissues and blood plasma samples

Human tissue and plasma samples from patients with metastatic BC were provided by the University Hospital Muenster (Ethik-Kommission der Ärztekammer Westfalen-Lippe und Universität Muenster; file number 2020-797-f-S and 2020-172-b-S). Informed consent was obtained from all subjects. The material comprises formaldehyde-fixed and paraffin-embedded bone stamp biopsy samples of female patients (luminal A-like, luminal B-like and TNBC subtypes). Plasma samples derived from BC patients with or without bone metastases and DCIS/LCIS controls.

### Animal experiments

All mice were kept under standard conditions, 22–23 °C with humidity of 50–60% and 12:12 h interval lighting in individually ventilated cages (IVC). All animal experiments were approved by the local authorities (Landesamt für Natur-, Umwelt- und Verbraucherschutz; LANUV; permits 84-02.04.2019.A184) and conducted in accordance with the Guide for Care and Use of Laboratory Animals.

#### Syngeneic 4T1 breast mouse model

Ultrasound was used for visualization of the heart to inject 5× 10^5^ luciferase labelled 4T1 tumor (4T1-Luc2, ATCC) cells into the left ventricle of 4-week-old female BALB/c mice [48]. The animals received one dose of analgesic (Carprofen, 5 mg/kg BW, 50 μl, s.c.) 30 min prior to tumor cell implantation, followed by two doses of analgesic at 24 h intervals. After a recovery period of four days, mice were injected intraperitoneally (i.p) with 50 mg/kg of Mstn antibody RK35 (Pfizer) or vehicle control every second day for a period of two weeks. During the experimental period, animals were monitored regularly and bioluminescence imaging (MS FX PRO, Bruker BioSpin MRI GmbH) was performed twice weekly. Initially, an injection of 4 mg luciferin was i.p administered. For a better spatial assignment of the bioluminescence (BLI) signal, an X-ray image was taken. Mice were euthanized on day 14.

#### Xenograft MDA breast cancer mouse model

4-week-old female NOD scid gamma mice (NSG, Charles River) were injected with 5×10^3^ human luciferase-labelled MDA cells (MDA-MB-231 Luc2, Caliper) into the tail artery [46]. After a recovery period of seven days, mice were i.p injected with 50 mg/kg anti-Mstn RK35 (Pfizer) or vehicle control twice weekly for a period of two weeks. Mice were euthanized on day 21.

#### Bioluminescence imaging (BLI)

BLI imaging was conducted using a MS FX PRO, Bruker BioSpin MRI GmbH. Imaging was performed under general isoflurane inhalation anesthesia and 4 mg luciferin was i.p. administered ~10 min before imaging. Measurements were taken in both dorsal and ventral positions, for 1 or 5 min based on the BLI signals of the injected cells. For a better spatial assignment of the BLI signal, an additional X-ray of the animals was taken. Quantification of the signal was performed using Bruker software.

### Micro-CT analysis

Bones were fixed in 4% PFA for 24 h and washed with PBS. The proximal tibial metaphysis and mid diaphysis were scanned with a resolution of 9 µm using SkyScan 1176 system (Bruker). The following settings were used: X-ray voltage was 45 kVp and 555 µA, respectively. Beam hardening was reduced using an aluminum filter and a mean of 5 pictures was taken at each angle (0.3°) to generate final images.

Data were reconstructed and analyzed by using different software packages from Bruker: NRecon v1.6.10.4 (Bruker), Data viewer, CT Analyzer v.1.16.4.1 (Bruker). Samples were shaded in MeshLab v2016.12 (Visual Computing Laboratory) using ambient occlusion and snapshots were taken.

### Histology

Tissues were fixed in 4% paraformaldehyde (PFA) overnight, decalcified in 10% EDTA/ TBS + 1% PFA and embedded in paraffin and sectioned into 5 μm slices. For OC visualization, a TRAP Kit (Sigma) was used and TRAP+ cells were counted in a 1500 µm^2^ defined area underneath the growth plate. For H&E staining, paraffin sections were stained with hematoxylin for 2.5 min. Sections were blued under tap water for 15 min and stained with eosin for a further 5 min. For immunohistochemical staining, tissue sections were pre-treated with citrate buffer pH 6.0 for 10 min at 95 °C, blocked with 5% horse serum and incubated with human/mouse/rat GDF-8/Myostatin antibody C-terminal region (R&D Systems, AF788, 1:50) overnight at 4 °C. Slices were incubated with secondary biotinylated antibody (Vector Laboratories, Burlingame, CA), streptavidin-biotin enzyme complex from the ABC kit (Vectastain) and DAB substrate (3,39-diaminobenzidine tetrahydrochloride) (Sigma). Hematoxylin was used for counterstaining (Vector Laboratories, Burlingame, CA). For p-SMAD2 antigen retrieval was performed using citrate buffer (pH 2.5) for 2 h at RT, blocked with 10% fetal calf serum (FCS) for 1 h at RT and incubated with antibody against p-SMAD2 (Ser465/467) (138D4) rabbit mAb (Cell Signaling Technology; 1:150) overnight at 4 °C. Sections were incubated with an HRP-conjugated polyclonal goat anti-rabbit secondary antibody (Vector Laboratories, Burlingame, CA) and immunodetection was performed using ImmPACT AMEC Red HRP substrate (Vector Laboratories, Burlingame, CA). Sections were counterstained with methyl green, rinsed in distilled water, and mounted using Moviol. Negative controls were included using rabbit IgG.

### In vitro osteoclast differentiation

Bone marrow derived macrophages (BMDMs) were isolated from femora and tibiae of 10- to 12- weeks old WT mice. Bones were opened at the corresponding joint and centrifuged at 4000 rpm and 30 s for cell isolation. 1 × 10^5^ BMDMs were then placed into a 96-well plate and cultured with alpha-MEM ( + 10% fetal bovine serum, 1% penicillin/streptomycin) supplemented with 30 ng/ml M-CSF for 3 days followed by stimulation with 30 ng/ml M-CSF (R&D) and 50 ng/ml RANKL (R&D) for further 4-6 days. Mature OCs were evaluated using TRAP staining kit (Sigma). The entire wells were recorded with the Axio Observer Z1 (ZEISS) and OC number as well as size of OCs with > 3 nuclei were measured by ZEN blue software.

### Co-culture and conditioned media experiments

For OC differentiation, 2 × 10^5^ BMDMs were seeded into a 96-well plate and pre-differentiated in alpha-MEM with 30 ng/ml M-CSF for 3 days. From day four on, 10% CM of the cancer cells or inhibitors such as anti-Mstn antibody (5 μg/ml) (R&D) were added along with 50 ng/ml RANKL and 30 ng/ml M-CSF. After another three days, the medium was changed and the experiment was stopped after 7–9 days. For (BMDMs+BC cells) co-culture experiments, 1 × 10^5^ BMDMs were seeded into a 96-well plate in alpha-MEM with 30 ng/ml M-CSF. After the cells were allowed to settle, either 25 4T1 cells or 50 MDA cells were added per well and cultured for 3 days with 30 ng/ml M-CSF. From day 4 on 30 ng/ml M-CSF and 50 ng/ml RANKL were added. After another three days, the medium was changed and the experiment was stopped after 7–9 days. Mature OCs were evaluated using TRAP staining kit (Sigma). The entire wells were recorded with the Axio Observer Z1 (ZEISS) and OC number as well as size of OCs with > 3 nuclei were measured by ZEN blue software.

### Immunofluorescence staining

For OC differentiation, 2 × 10^6^ BMDMs were seeded into a 24-well plate with glass cover slips and pre-differentiated in alpha-MEM with 30 ng/ml M-CSF for 3 days. From day four on 50 ng/ml RANKL and 30 ng/ml M-CSF were added for 2 days. At day 5, 10% CM of the cancer cells or inhibitors such as anti-Mstn antibody (5 μg/ml) (R&D) were added for 1 hour. Following differentiation, cells were washed with PBS and fixed with 4% PFA (pH 7.4) for 20 min. In a next step, cells were quenched with 100 mM NH_4_Cl. The cells were then permeabilised with 0.5% Triton-X100 in PBS, followed by PBS washes. Next, cells were incubated for an hour in a blocking solution containing 10% normal horse serum in PBS and stained overnight at 4 °C with a primary p-SMAD2 antibody ((Ser465/467) (138D4) rabbit mAb, Cell Signaling Technology; 1:100). The next day, cells were washed and stained for 30 min in the dark with an Alexa Fluor 488-conjugated secondary antibody (Invitrogen #A11034, dilution 1:5000). Nuclear staining was performed using 4′,6-diamidino-2-phenylindole (DAPI) (Invitrogen #D1306, dilution 1:10,000), and F-actin cytoskeleton was visualized by Rhodamin-Phalloidin (Invitrogen #R415, dilution 1:5000). Finally, cells were washed with tap water and mounted with Mowiol (Sigma-Aldrich). Images were captured using a Zeiss AxioImager.M2 microscope equipped with ZEN 3.2 software (Zeiss, Jena, Germany). Quantification of relative fluorescence intensity of p-SMAD2 was calculated using Fiji (ImageJ) software.

### In vitro bone resorption assay

Bone resorption assays were performed by seeding 6 × 10^5^ BMDMs on calcium phosphate coated 48-well plates (Hölzel) and 250 4T1 cells or 500 MDA cells were added followed by priming and stimulation as described for OC differentiation. After 12–14 days cells were removed by treating the wells with 5% sodium hypochlorite and washing three times with PBS. The entire wells were recorded with the Axio Observer Z1 and number and area of resorption pits were quantified by using ImageJ software.

### SDS PAGE and western blotting

Total cell extracts were obtained using NP-40 buffer [150 mM sodiumchloride, 1% NP-40, and 50 mM tris-HCl (pH 8)] containing protease inhibitor cocktail (Roche) and phosphatase inhibitor (Sigma). The extracts were resolved by SDS–polyacrylamide gel electrophoresis (PAGE) and transferred to a polyvinylidene difluoride membrane (GE Healthcare). The proteins were detected with appropriate antibodies using the ECL detection system (GE Healthcare). Antibodies against the following proteins were used: GAPDH (Cell Signaling), anti-Mstn (AF788, R&D), p-SMAD2 ((Ser465/467) (138D4) rabbit mAb, Cell Signaling Technology and total SMAD2 (SMAD2 (D43B4) XP® Rabbit mAb).

Formalin fixed skeletal muscle tissue samples were snap-frozen in liquid nitrogen and subsequently stored at –80 °C until processing. For homogenization, the frozen muscle samples were pulverized using a cryomill operated at a frequency of 30 Hz for 1 min and 30 s. The resulting muscle powder was resuspended in 500 µl of PEB buffer (300 mM Tris base, 2% SDS, pH 8.0) and thoroughly vortexed to ensure complete mixing. Samples were then incubated at 90 °C for 90 min to facilitate protein extraction. After incubation, the samples were centrifuged at 16,000 g for 20 min to pellet insoluble debris. For Western blot analysis, 10 µl of the protein extract was mixed with 10 µl of PEB buffer and 4 µl of loading buffer before electrophoresis. The protocol was adapted from Kawashima et al. [49].

### ELISA

Blood plasma from BC patients was analyzed using the human/mouse/rat GDF-8/Myostatin ELISA kit (R&D Systems, #DGDF80), according to the manufacturer’s instructions.

### Quantitative real-time PCR (qRT-PCR)

Total RNA was isolated from the cells using the High Pure RNA isolation kit (Roche) and 1 µg of RNA was reversely transcribed into cDNA (iScript cDNA synthesis kit, Bio-Rad). The qRT-PCR analysis was performed in triplicates with 10 ng of cDNA per reaction. Gene expression was measured by SYBR green detection on a QIAquant® 384 (Qiagen). *GNB2L1* and *HPRT1* served as housekeeping genes. Primer sequences are listed in Supplementary Table 3.

All antibodies, recombinant proteins, commercial assays, cell lines, experimental models and software used in this study are listed in Supplementary Table 4.

### Statistics

Statistical significance was calculated by one-way ANOVA for multiple comparisons or Mann-Whitney U test. Statistical significance was analyzed in in vivo and in vitro data from at least three independent experiments. *p < 0.05, **p < 0.01, ***p < 0.005 and ****p < 0.0001 were defined as significant. All data are presented as mean ± SEM unless otherwise specified. All samples were randomized and analyzed blinded.

## Supplementary information

Supplementary material

## Data Availability

All data generated or analyzed during this study are included in this published article.
