# Supplementary material for: Pharmacological inhibition of myostatin effectively ameliorates osteolytic lesions in syngeneic and xenograft breast cancer mouse models
Source: Oncogene. 2025 Nov 17;44(49):4781–95. doi: 10.1038/s41388-025-03622-7 (PMC12657228; doi:10.1038/s41388-025-03622-7)
Supplement: Supplementary file 1 — Supplementary material [file 41388_2025_3622_MOESM1_ESM.pdf]

Supplementary figures

Suppl.Fig.1

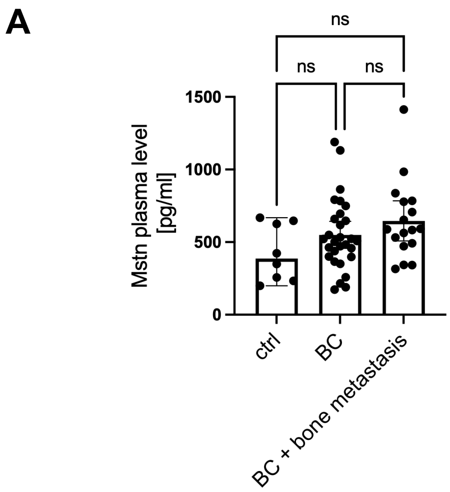

Suppl.Fig. 1: Systemic Mstn levels in breast cancer patients with and without bone metastasis. Mstn levels were measured by ELISA in plasma samples from DCIS/LCIS control patients (ctrl), n=8; breast cancer patients without bone metastasis (BC) n=29; breast cancer patients with bone metastasis (BC+bone metastasis) n=17. All data are median ± 95% CI, one-way ANOVA, Kruskal-Wallis test with multiple comparisons.

Suppl.Fig.2

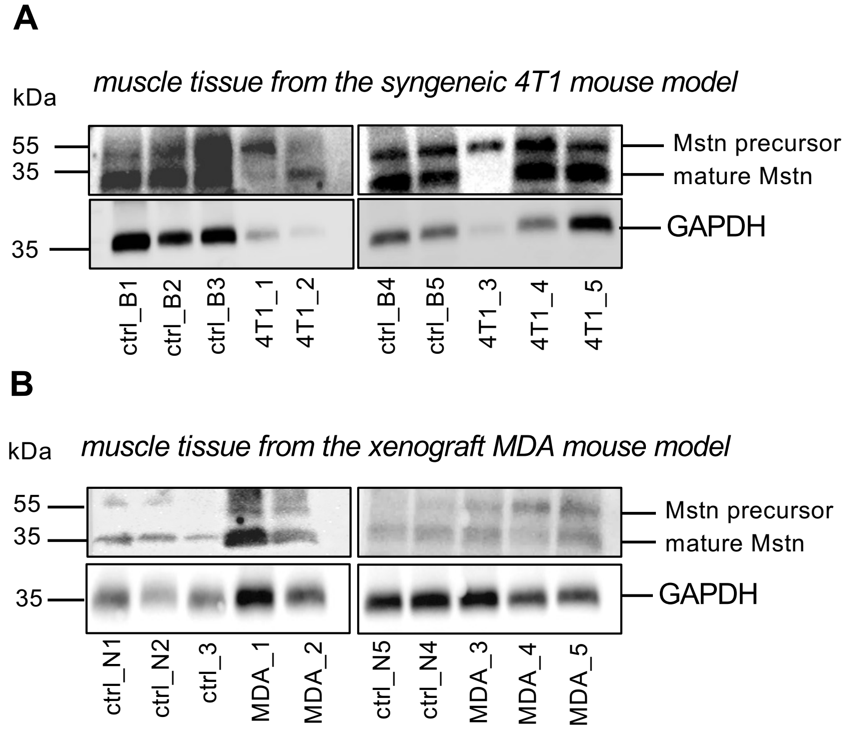

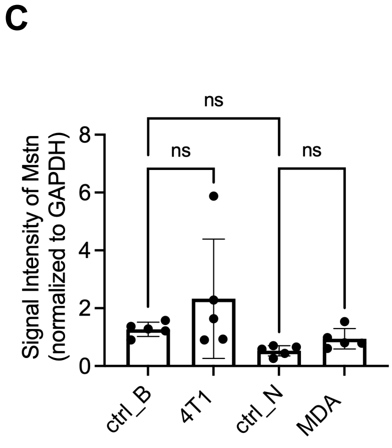

Suppl. Fig. 2: **Mstn expression in muscle tissue did not differ between naïve mice and those injected with MDA or 4T1 tumor cells.** (A-B) Mstn protein expression was analyzed by western blot using formaldehyde-fixed muscle tissues from the tibia and femur of mice. Controls B1-B5 (ctrl_B1-B5): naïve Balb/c mice; 4T1_1-5: 4T1-injected Balb/c mice; Controls N1-N5 (ctrl_N1-N5): naïve NSG mice; MDA_1-5: MDA-injected NSG mice. (n=5 mice each group). **C)** Signal intensity of was analyzed using Fiji software and normalized to GAPDH. All data are mean ± SD, one-way ANOVA, Kruskal-Wallis test with multiple comparisons, * $p \leq 0.05$, ** $p \leq 0.01$.

Suppl.Table 1: Formalin-fixed paraffin-embedded (FFPE) tissue samples from BC patients with bone metastasis for immunohistological analysis of Mstn. Cohort of 27 patients. Bone samples derived from femur, humerus, illiac crest, ilium, sacrum, lumbar vertebrae, clavicle, clivus. n/a: not applicable. wt: wild type, mut: mutant.

FFPE tissue samples from BC patients with bone metastasis		
**Parameter**	**Samples, n=27**	**Samples. n in %**
Gender (female)	27	100
Median age (years, range (years)	58 (37-93)	
**Origin of samples, n and %**		
Bone metastases	27	100
**Molecular Subtype, n and %**		
LuminalA-like	9	33,3
Luminal-B like	11	40,7
HER2-enriched	0	0,0
Triple-negative	7	25,9
**Menopausal status**		
perimenopausal	0	0,0
premenopausal	4	14,8
postmenopausal	17	63,0
n/a	6	22,2
BRCA1, **n and %**		
wt	2	7,4
mut	0	0,0
n/a	25	92,6
BRCA2, **n and %**		0,0
wt	2	7,4
mut	0	0,0
n/a	25	92,6
PIK3CA, **n and %**		
wt	0	0,0
mut	2	7,4
n/a	25	92,6
**Survival staus, n and %**		
Alive or unknown	24	88,9
Dead	3	11,1

Suppl. Table 2: Human blood plasma samples for analysis of Mstn expression using ELISA. Cohort of 54 patients. n/a: not applicable. wt: wild type, mut: mutant.

Blood plasma samples		
**Parameter**	**Samples, n=54**	**Samples. n in %**
Gender (female)	54	100
Median age (years, range (years)	60 (28-89)	
**Diagnosis, n and %**		
Primary BC tumor	29	53,7
Primary BC tumor + bone metastases	17	31,5
DCIS	7	13,0
LCIS	1	1,9
**Molecular Subtype, n and %**		
LuminalA-like	14	30,4
Luminal-B like	17	37,0
HER2-enriched	4	8,7
Triple-negative	11	23,9
**Menopausal status**		
perimenopausal	5	9,3
premenopausal	7	13,0
postmenopausal	40	74,1
n/a	2	3,7
BRCA1, **n and %**		
wt	14	30,4
mut	2	4,3
n/a	30	65,2
BRCA2, **n and %**		
wt	13	28,3
mut	3	6,5
n/a	30	65,2
PIK3CA**, n and %**		
wt	4	7,4
mut	3	5,5
n/a	47	87,0
**Survival staus, n and %**		
Alive or unknown	48	88,9
Dead	6	11,1

Suppl. Table 3: Primer used for qRT-PCR.

Gene name	Forward primer (5'-3')	Reverse primer (5'-3')
FST	CCTAAAGGCAAGATGTAAAGAG	CACAGTAGGCATTATTGGTC
FSTL3	GAACAAGATCAACCTCCTCG	CACGAATCTTTGCAGGGA
GDF-11	GACCTACACGACTTCCAG	TGCCATCTGTCTGTACTG
GNB2L1	AACCCTATCATCGTCTCCT	CAATGTGGTTGGTCTTCAG
HPRT1	TATGCTGAGGATTTGGAAAGG	CATCTCCTTCATCACATCTCG
INHBA	CAGACCTCGGAGATCATCAC	CCTTGGAAATCTCGAAGTGC
INHBB	GCGTTTCCGAAATCATCAG	GGAGTTTCAGGTAAAGCCA
MSTN	ATCTTGCTGTAACCTTCCCA	AGCATCGTGATTCTGTTGAG

Suppl. Table 4: Key Resources: all antibodies, recombinant proteins, commercial assays, cell lines, human samples, experimental models and software used in this study.

Reagent or Resource	Source	Identifier
**Antibodies**		
Mstn antibody-C-terminal region	Sigma-Aldrich, St. Louis, USA	Cat# AB3239
anti-Mstn	R&D Systems, Inc., Minneapolis, USA	Cat# AF788
Anti-GDF8 mRK35 antibody	Pfiizer, New York, USA	N/A
p-SMAD2 ((Ser465/467) (138D4) rabbit mAb	Cell signaling Technology, Danvers, Massachusetts, USA	Cat#3108
total SMAD2 (D43B4) XP® Rabbit mAb)	Cell signaling Technology, Danvers, Massachusetts, USA	Cat#5339
**Chemicals, Peptides, and Recombinant Proteins**		
Recombinant Human/Mouse/Rat GDF-8/Myostatin Protein	R&D Systems, Inc., Minneapolis, USA	Cat# 788-G8
Bone Resorption Assay Plate 48x2	Hoelzel Biotech, Cologne, Germany	Cat# CSR-BRA-48X2P
Human/Mouse/Rat GDF-8/Myostatin Antibody	R&D Systems, Inc., Minneapolis, USA	Cat# AF788
Recombinant Mouse TRANCE/ RANKL/ TNFSF11	R&D Systems, Inc., Minneapolis, USA	Cat# 462-TEC
Recombinant Mouse M-CSF Protein	R&D Systems, Inc., Minneapolis, USA	Cat# 416-ML
**Critical Commercial Assays**		
Acid Phosphatase, Leukocyte (TRAP) Kit	Sigma-Aldrich, St. Louis, USA	Cat# 387A
Vectastain ABC-AP Kit, Vector Red Substance	Vector Laboratories, Inc., Burlingame, CA, USA	Cat# AK-5000
DAB, Peroxidase (HRP)	Vector Laboratories, Inc., Burlingame, CA, USA	SK-4100
**Human samples**		
Human bone metastases tissue	Gerhard-Domagk-Institute for Pathology, University of Muenster, Germany	
Human plasma samples	Department of Medicine A /Department of Gynecology and Obstetrics, University of Muenster, Germany	
**Experimental Models: Cell Lines**		
4T1/ 4T1-Luc2	ATCC, Virginia, USA	Cat# CRL-2539-LUC2™
MDA-MB-231/MDA-MB-231-Luc2	ATCC Virginia, USA /Caliper Life Science, Massachusetts, USA	
HS578T	DSMZ, Braunschweig, Germany	Cat# ACC 781
HCC1143	DSMZ, Braunschweig, Germany	Cat# ACC 517
HCC-1937	DSMZ, Braunschweig, Germany	Cat# ACC 513
MDA-MB-468	DSMZ, Braunschweig, Germany	Cat# ACC 738
**Experimental Models: Organisms/Strains**		
Balb/c AnCrl-Mouse	Charles River laboratories	https://www.criver.com
NSG (NOD SCID gamma)	Charles River laboratories	https://www.criver.com
**Software and algorithms**		
Prism 9 for Mac OS X, Version 9.4.0	GraphPad Software, Inc, San Diego, USA	https://www.graphpad.com/scientific-software/prism/
ImageJ v1.52a	Wayne Rasband, National Institutes of Health, USA	https://imagej.nih.gov/ij/
ZEN 2 v.2.0.0.0	Zeiss, Oberkochen, Germany	https://www.zeiss.com/
SkyScan 1176 high resolution in vivo microtomograph (µCT)	Bruker, Billerica, USA	https://www.bruker.com/
Nrecon v1.6.10.4	Bruker, Billerica, USA	https://www.bruker.com/
CT Analyser v.1.16.4.1	Bruker, Billerica, USA	https://www.bruker.com/
DataViewer v1.5.2.4	Bruker, Billerica, USA	https://www.bruker.com/
Meshlab v2016.12	Visual Computing Laboratory, Pisa, Italy	https://www.meshlab.net
MS FX PRO	Bruker BioSpin MRI GmbH	https://www.bruker.com/
